# Recent evolutionary history predicts population but not ecosystem‐level patterns

**DOI:** 10.1002/ece3.5879

**Published:** 2019-11-28

**Authors:** Madison L. Miller, John A. Kronenberger, Sarah W. Fitzpatrick

**Affiliations:** ^1^ W. K. Kellogg Biological Station Michigan State University Hickory Corners MI USA; ^2^ National Genomics Center for Wildlife and Fish Conservation USDA Forest Service Missoula MT USA; ^3^ Department of Integrative Biology Michigan State University Hickory Corners MI USA

**Keywords:** eco‐evolutionary dynamics, gene flow, genetic drift, genetic rescue, population dynamics

## Abstract

In the face of rapid anthropogenic environmental change, it is increasingly important to understand how ecological and evolutionary interactions affect the persistence of natural populations. Augmented gene flow has emerged as a potentially effective management strategy to counteract negative consequences of genetic drift and inbreeding depression in small and isolated populations. However, questions remain about the long‐term impacts of augmented gene flow and whether changes in individual and population fitness are reflected in ecosystem structure, potentiating eco‐evolutionary feedbacks. In this study, we used Trinidadian guppies (*Poecilia reticulata*) in experimental outdoor mesocosms to assess how populations with different recent evolutionary histories responded to a scenario of severe population size reduction followed by expansion in a high‐quality environment. We also investigated how variation in evolutionary history of the focal species affected ecosystem dynamics. We found that evolutionary history (i.e., gene flow vs. no gene flow) consistently predicted variation in individual growth. In addition, gene flow led to faster population growth in populations from one of the two drainages, but did not have measurable impacts on the ecosystem variables we measured: zooplankton density, algal growth, and decomposition rates. Our results suggest that benefits of gene flow may be long‐term and environment‐dependent. Although small in replication and duration, our study highlights the importance of eco‐evolutionary interactions in determining population persistence and sets the stage for future work in this area.

## Graphical abstract

We used the model Trinidadian guppy system to investigate how evolutionary history (i.e., drift vs. gene flow) impacts individual and population growth rates in a novel environment. We also tested whether evolutionary history affects ecosystem variables. Our results suggest that a history of gene flow increases individual and population growth, but does not necessarily scale to the ecosystem level.

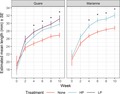

## 1 INTRODUCTION

Evolution and ecology were historically assumed to operate on different timescales (Slobodkin, 1961). However, empirical evidence increasingly shows that evolutionary processes can occur quickly enough to influence contemporary population, community, and ecosystem dynamics (Carroll, Hendry, Reznick, & Fox, 2007; Hairston, Ellner, Geber, Yoshida, & Fox, 2005; Harmon et al., 2009; Yoshida, Jones, Ellner, Fussmann, & Hairston, 2003). Most research in the field of eco‐evolutionary dynamics focuses on evolution via natural selection (Lowe, Kovach, & Allendorf, 2017). For example, Darwin's finches were famously shown to undergo rapid evolution in beak size in response to seed size availability, which is correlated with rainfall patterns (Grant, 1986). In addition to many examples of adaptation on ecological timescales, increasing empirical evidence shows how adaptive differentiation in one species can cause measurable effects on environmental parameters (Ezard, Côté, & Pelletier, 2009; Pelletier, Garant, & Hendry, 2009). A mesocosm study showed that recently diverged benthic and limnetic threespine stickleback morphs had quantifiable effects on prey community structure, primary production, and the nature of dissolved organic materials (Harmon et al., 2009). Another study found that phenotypically divergent alewives significantly changed the structure of their zooplankton prey communities, specifically altering mean body size, total biomass, species richness, and diversity (Post & Palkovacs, 2009). However, less is known about the ecological role of nonselective evolutionary forces such as genetic drift and gene flow. Addressing both selective and nonselective forces is necessary to understand the full scope of eco‐evolutionary interactions (Lowe et al., 2017).

Evaluating how changes in evolutionary processes affect demography is vital for predicting population persistence, especially under rapid environmental change (Lande, 1988). Small populations are vulnerable to Allee effects and inbreeding, the latter of which can lead to inbreeding depression and loss of genetic variation (Kinniston & Hairston, 2007). These “small population problems” can constrain adaptation under novel selection pressures and may contribute to an “extinction vortex,” the positive feedback loop of environmental and biological interactions that causes population decline and eventual extinction (Gilpin & Soule, 1983). For example, shorter life spans and lower larval survival rates in inbred Glanville fritillary butterfly populations increased their extinction risk (Saccheri et al., 1998). On the other hand, some populations and taxa persist in spite of small population sizes. For instance, the Apennine brown bear population in Italy has survived for many generations despite being small, inbred, and having startlingly low levels of genetic variation (Benazzo et al., 2017). Given contradicting examples like these, more research is needed to determine the eco‐evolutionary predictors of population dynamics and extinction risk.

Gene flow is a nonselective evolutionary force that can mitigate small population problems by reversing the effects of inbreeding depression and genetic drift. By increasing genetic variation, gene flow can also facilitate a faster response to natural selection (Swindell & Bouzat, 2006). Gene flow manipulations are an emerging management strategy in which a small number of immigrants are translocated into a (typically) small, inbred population in an effort to restore connectivity among recently isolated populations and ultimately increase population fitness (Aitken & Whitlock, 2013; Tallmon, Luikart, & Waples, 2004; Whiteley, Fitzpatrick, Funk, & Tallmon, 2015). Genetic rescue, an increase in population growth caused by gene flow, has been shown to produce large and consistent benefits to population fitness (Frankham, 2015; Whiteley et al., 2015). Several iconic conservation success stories resulting from genetic rescue have been documented in species such as Florida panthers (Johnson, Chappell, et al., 2010), greater prairie chickens (Westemeier et al., 1998), and bighorn sheep (Hogg, Forbes, Steele, & Luikart, 2006). Although examples like these suggest that assisted gene flow is an effective management tool, it has not been widely used due to concerns of outbreeding depression, genetic swamping, and disease introduction (Edmands, 2007; Frankham, 2015).

While increasing evidence suggests that gene flow into genetically depauperate populations in the wild can boost genetic variation and thereby increase individual and population fitness (Fitzpatrick et al., 2016; Robinson et al., 2017; Weeks et al., 2017), little is known about how recent episodes of gene flow versus genetic drift affect demography under environmental change. If the environment is poor, gene flow, even if beneficial, may not manifest in higher population sizes. For example, immigration of a single outbred wolf into the isolated Isle Royale population led to an initial increase in heterozygosity without subsequent increases in population size, an outcome thought to be due to poor environmental conditions (Adams, Vucetich, Hedrick, Peterson, & Vucetich, 2011). Conversely, it has not been empirically tested whether populations that have experienced recent gene flow are better able to take advantage of good quality environments than those experiencing high genetic drift. Understanding how recent evolutionary history determines persistence and adaptive potential under changing environments is urgently important given current levels of habitat destruction, climate change, and anthropogenic disturbance facing natural populations today (Chevin, Lande, & Mace, 2010). It is essential to understand how manipulating evolutionary forces affects population demography and persistence and specifically how human‐assisted gene flow may impact the surrounding environment.

Genetic rescue is inherently eco‐evolutionary because it involves manipulating an evolutionary force, gene flow, to affect the dynamics of populations. However, it is unknown the extent to which evolutionary manipulations scale up to affect ecosystem dynamics. There is reason to believe that there would be an effect on ecosystem dynamics since genetic rescue often causes dramatic increases in abundance of the “rescued” population (Fitzpatrick et al., 2016). For example, trophic cascades could be affected if increased abundance of a top predator results in higher depletion rates of its primary prey species. Additionally, populations adapted to dissimilar environments may use resources differently and cause significant ecosystem structure alterations in short time periods (Bassar et al., 2010). If immigration from an adaptively divergent population occurs, the ecological role of the recipient population may be altered. Finally, levels of intraspecific genetic variation can impact the ecological role of populations (Hughes, Inouye, Johnson, Underwood, & Vellend, 2008). For example, different levels of population genetic variation in tall goldenrod, *Solidago altissima*, were shown to predict arthropod diversity and community structure (Crutsinger et al., 2006). Linking changes in recent evolutionary history to changes in population genetic variation to changes in the ecosystem has not been done experimentally. Understanding such links has important consequences for biodiversity conservation under rapid environmental change.

Trinidadian guppies are a model system frequently used in eco‐evolutionary research due to short generation times (3–4 per year; Magurran, 2005; Reznick & Endler, 1982), rapid adaptation in new environments (Magurran, 2005), and predictable phenotypic traits like offspring size, coloration, and life histories based on the environment (Ghalambor, McKay, Carroll, & Reznick, 2007). Streams that drain the Northern Range Mountains in Trinidad have varying predator assemblages, imposing selection pressures that have resulted in the repeated evolution of divergent guppy life histories. Guppies from high predation (HP) environments, found in lower‐elevation stream reaches, have faster maturation rates and more, smaller offspring than guppies found in low predation (LP) environments at higher elevations (Reznick & Endler, 1982). Headwater populations of guppies also typically have lower genetic variation and smaller population sizes due to founder effects caused by geographical barriers such as waterfalls (Barson, Cable, & Oosterhout, 2009). Previous work shows that inbred guppies often have lower fitness than outbred guppies due to higher susceptibility to parasite infection (Smallbone, Oosterhout, & Cable, 2016), less coloration, fewer mating behaviors (Van Oosterhout et al., 2003), and fewer offspring produced (Johnson, Onorato, et al., 2010). Studies documenting fitness effects of gene flow manipulation in wild populations showed that immigration dramatically increased genetic diversity and population sizes, without the loss of locally adapted traits (Fitzpatrick et al., 2016; Fitzpatrick, Gerberich, Kronenberger, Angeloni, & Funk, 2015).

**Figure 1:**
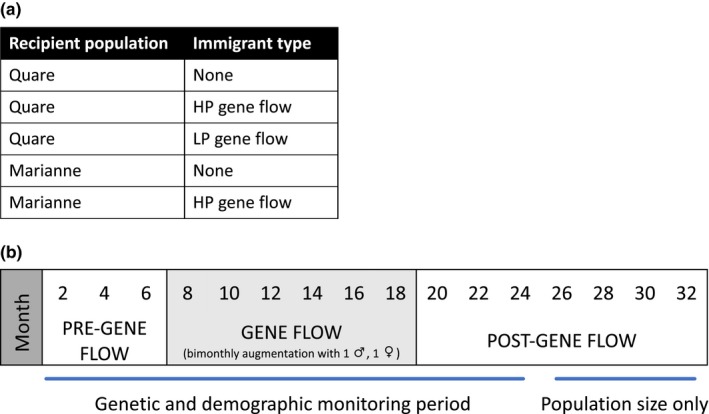
Guppies in the present study were sampled from subset of gene flow treatments from Kronenberger et al. (2018) (a) where recipient populations received either no augmentation (None), gene flow from a high predation (HP) guppy population, or gene flow from a low predation (LP) guppy population. Populations were censused bimonthly for a total of 32 months with a 6‐month pre–gene flow period, 12‐month gene flow augmentation period, and 14‐month post–gene flow period (b). Guppies at month 32 were used to seed mesocosm populations in the present study

This study builds on a previous experiment that tested fitness effects of gene flow in replicated populations housed in glass aquaria in a laboratory environment (Kronenberger et al., 2018). Kronenberger et al. (2018) documented strong and consistent evidence for genetic rescue in a recipient population (Quare) that had initially lower levels of genetic variation, but inconsistent effects of gene flow in a second recipient population (Marianne) that started with higher levels of genetic variation. Initial differences between the two recipient populations in heterozygosity (Quare *H*_e_ = 0.71; Marianne *H*_e_ = 0.87) and effective population size (Quare *N*_e_ = 247; Marianne *N*_e_ = 949) suggested that Quare guppies experienced stronger genetic drift than Marianne, and therefore, stood to benefit more from gene flow initially. Laboratory populations from this experiment were maintained for more than two years (about six to eight guppy generations) after the immigration treatments began (Figure 1).

We used the descendants of the aforementioned experimental populations to test whether recent evolutionary history (i.e., gene flow versus no gene flow treatments from Kronenberger et al. (2018)) predicted individual growth and population growth rates in novel, high resource environments. We hypothesized that guppy populations that have experienced recent gene flow would show faster individual and population growth rates and attain larger population sizes compared to populations without gene flow due to added genetic variation. We also tested whether ecosystem dynamics varied between populations with different recent evolutionary histories. If populations that received gene flow exhibited faster individual and population growth rates, we expected resources to be depleted more quickly in those environments. We also predicted that populations with higher genetic variation due to recent gene flow might utilize a more diverse set of resources than populations with lower genetic variation. This is the first study we know of to test ecosystem‐level consequences of gene flow manipulation. Results of this experiment enhance understanding of eco‐evolutionary interactions by shedding light on how differences in nonadaptive processes, such as gene flow and genetic drift, can influence demography and ecosystem dynamics in changing environments.

## 2 METHODS

### 2.1 Mesocosm setup

Our experiment took place at Michigan State University's W. K. Kellogg Biological Station Experimental Pond Facility from June 2017 to October 2017. We acid‐washed and filled 24 cattle tanks (1,000 L) with well water and added sand substrate. To create high resource pond communities, we collected a total of 12.5 L of zooplankton‐rich water using a plankton tow net from four nearby ponds and inoculated each tank with 500 ml of this mixture to provide a prey resource for the guppies. We also added 15 g of a common aquatic plant species, hornwort (*Ceratophyllum demersum*), to each tank to provide cover for young fish from cannibalistic adults. All tanks received a nutrient mixture to increase primary productivity; 10.4 g nitrogen (NaNO_3_) and 0.33 g phosphorus (NaH_2_PO_4_) was dissolved in 50 ml of water and added to each tank (Rudolf & Rasmussen, 2013). We added six (4.5 cm^2^) ceramic tiles to each tank to measure the accumulation of benthic algae. To compare decomposition rates between treatments, 10 × 15 cm mesh bags were filled with 7 g of air‐dried mixed leaf litter collected from the W.K. Kellogg Bird Sanctuary and submerged in each tank.

### 2.2 Experimental design

The guppies used in our experiment initially were collected from LP tributaries of the Quare and Marianne rivers in Trinidad and Tobago, as described in Kronenberger et al. (2018). In that experiment, populations were established in 38 L glass aquaria with 16 guppies of a 1:1 sex ratio. Following a six‐month period without gene flow, populations were augmented with one male and one female wild‐caught immigrant bimonthly for 12 months. Genetic and demographic monitoring continued for 6 months post–gene flow and reduced monitoring of abundance only continued an additional eight months (Figure 1). Following conclusion of the full 32‐month gene flow study, subsets of these populations were moved to the MSU Experimental Pond Facility for our experiment in July 2017 (Figure 1). Our experimental populations were comprised of Kronenberger et al. (2018) fish from three evolutionary history treatments across two populations: Quare with no prior gene flow, Marianne with no previous gene flow, Quare with previous gene flow from a HP source population, Marianne with previous gene flow from a HP source population, Quare with previous gene flow from a LP source population (designated “LPF” in Kronenberger et al., 2018), and tanks with no fish to provide a control for ecosystem measurements. Fish were not available for a Marianne LP gene flow treatment. To initiate our experiment, two adult male and two size‐matched female guppies from each evolutionary history treatment were anesthetized with a dilute solution of MS‐222, measured for standard length (distance from tip of snout to tip of hypleural plate), marked with a unique visible implant elastomer pattern (VIE; Northwest Marine Technology, Inc.), and added to the cattle tanks after recovering in an aerated tank. Previous studies show low mortality and high recapture success using these methods (Reznick, Butler, Rodd, & Ross, 1996).

### 2.3 Data collection

We compiled the following data from each tank on a biweekly schedule from late July 2017 to early October 2017 (10 weeks total). Using a depth‐integrated tube sampler, we collected 4 L of water from each tank and filtered it through 75 µm mesh to determine zooplankton density. The samples were immediately stored in ethanol and later visualized under a dissecting microscope to calculate copepod and cladoceran density (individuals/L). We extracted one tile of the six total tiles from each tank every other week and weighed the oven‐dried algae present to calculate benthic algae accumulation. To quantify primary productivity, pelagic algae were collected by filtering 50–100 ml of water from each tank and storing algal samples on folded papers in foil at −20℃. These samples were later analyzed with a UV/VIS spectrophotometer to quantify the amount of chlorophyll‐a (μg/L) in each. We calculated decomposition rates by comparing the dry leaf litter mass (g) before and after the addition of mesh bags containing leaf litter. To ensure that water conditions were held constant between tanks throughout the course of our experiment, every other week we measured dissolved oxygen, temperature, and pH three times over a 24‐hr time period in all tanks. All guppies ≥ 14 mm were censused biweekly. New recruits (≥14 mm) were given unique elastomer marks, and all individuals were processed as described above to census populations and determine individual growth.

### 2.4 Data analysis

#### 2.4.1 Individual growth

We were interested in whether differences in recent evolutionary history led to differences in the rate of growth among individuals in a high resource environment. We only compared growth (measured as standard length) among founding females to avoid confounding juvenile with adult growth and female with male growth, the latter because female guppies have indeterminate growth throughout their lifetimes whereas male growth ceases at maturity. We estimated standard lengths at each sampling occasion using a generalized linear mixed model implemented with “proc glimmix” in SAS version 9.4 (SAS Institute). Residuals were not normally distributed, so we assumed a lognormal distribution as it was superior following a comparison of the model under all available distributions using Akaike Information Criterion corrected for small sample size (AIC_C_; Burnham & Anderson, 2002). Fixed effects included the three‐way interaction between population (Quare and Marianne), treatment (no gene flow, HP gene flow, and LP gene flow), and sampling occasion (week), in addition to all two‐way interactions and main effects. To account for nonindependence resulting from measuring the same individuals over time, we included sampling occasion as repeated random effect with a heterogeneous autoregressive covariance structure, in which the correlation between sampling occasions decreases exponentially as the interval between them increases. This covariance structure fits our expectation of how sampling occasions are related and was best supported according to AIC_C_ comparisons.

#### 2.4.2 Population growth

To assess whether there were changes in population fitness arising from recent evolutionary history, we used census population sizes to estimate population sizes over time and finite population growth rates of adult fish. Population sizes were estimated using a generalized linear mixed model with a Poisson distribution due to non‐normally distributed residuals, the typical distribution for nonoverdispersed count data. As in the individual growth model, fixed effects included the three‐way interaction between population, treatment, and sampling occasion in addition to all two‐way interactions and main effects. We modeled sampling occasion as a repeated random effect with an autoregressive covariance structure as above, although in this case, the covariance structure was not heterogeneous due to convergence issues. Finite population growth rates were calculated as the total number of individuals in each tank at the end of the experiment divided by 4 (the number of founding individuals) and estimated using multiple linear regression. Fixed effects included the population and treatment main effects and their interaction. Both models (population size and population growth rate) were run using “proc glimmix” in SAS version 9.4.

#### 2.4.3 Ecosystem effects

We used linear mixed models to test our predictions about how recent evolutionary history impacts environmental variables (decomposition rates, algal growth, and zooplankton density). For all ecosystem measurements, data from the last sampling period were analyzed and compared among treatments to elucidate the greatest differences between treatments. Models included treatment type (no gene flow, HP gene flow, LP gene flow, and control) as a fixed effect and tank location as a random effect. Each treatment was tested against the null model that included only the random effect. We also tested whether populations (Quare and Marianne) determined ecosystem structure and used linear models for each environmental variable. We used simple linear regressions to correlate adult fish abundance and total fish biomass with each environmental variable. All models were run using the “lme4” package in R version 3.5.1 (Bates & Maechler, 2009).

## 3 RESULTS

### 3.1 Individual growth

**Figure 2:**
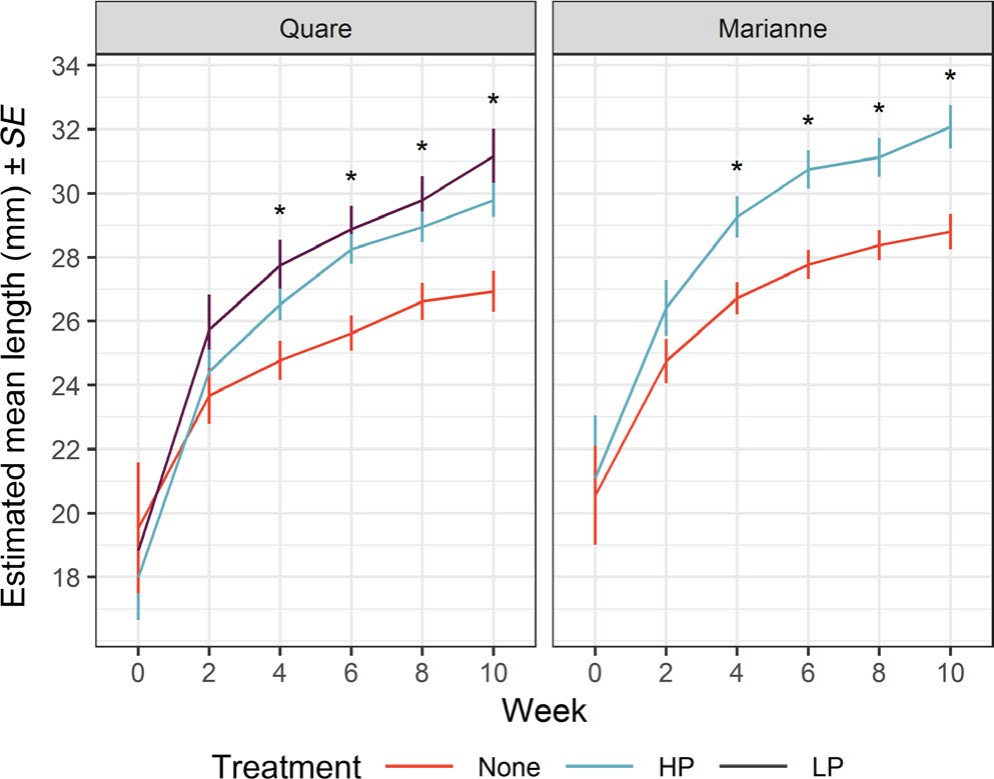
Estimated marginal means and standard errors of standard length, measured from founding females at the start of the experiment and on each subsequent sampling occasion. Colors correspond to evolutionary history treatments: red, no gene flow (none); blue, gene flow from a high predation source (HP); purple, gene flow from a low predation source (LP). Significant differences (*p* < .05) are indicated with asterisks; in all cases, HP and LP treatments were similar to one another but significantly different than the none treatment

Females from all treatments grew larger over the course of the experiment. However, females from populations that previously received gene flow grew faster than females from populations without gene flow (Table 1; Figure 2). Pairwise comparisons of estimated marginal means (i.e., means accounting for model structure) showed individuals from HP and LP gene flow treatments to be significantly larger than individuals from populations without recent gene flow. This pattern began in week four and lasted until the end of the experiment at week 10. Differences between the recent gene flow and no gene flow treatments were highly significant (*t*_101_ ≥ 2.98, *p* ≤ .0036) in all cases except for the Quare HP versus no gene flow treatment comparison, which was moderately significant (*t*_101_ = 2.19, *p* = .0309).

**Table 1:** The results of type 3 tests of fixed effects from our individual growth (standard length) and population growth (population size and finite population growth rate) models. Standard length and population size were modeled as repeated measures, generalized linear mixed models, and population growth rate was modeled as a multiple linear regression (see text for more details). For each model, we report numerator and denominator degrees of freedom and *F* and *p* values

Response	Fixed effect	Num. *df*	Den. *df*	*F*	*p*
Standard length	Population	1	21	7.99	.0101
	Treatment	2	21	3.56	.0467
	Population*Treatment	1	21	0.30	.5872
	Period	5	101	29.40	<.0001
	Population*Period	5	101	1.09	.3720
	Treatment*Period	10	101	1.01	.4438
	Population*Treatment*Period	5	101	0.16	.9761
Population size	Population	1	15	4.40	.0534
	Treatment	2	15	0.70	.5137
	Population*Treatment	1	15	5.02	.0406
	Period	5	75	17.64	<.0001
	Population*Period	5	75	0.98	.4340
	Treatment*Period	10	75	0.50	.8861
	Population*Treatment*Period	5	75	1.32	.2632
Population growth rate	Population	1	15	1.45	.2478
	Treatment	2	15	0.89	.4315
	Population*Treatment	1	15	1.00	.3322

### 3.2 Population growth

**Figure 3:**
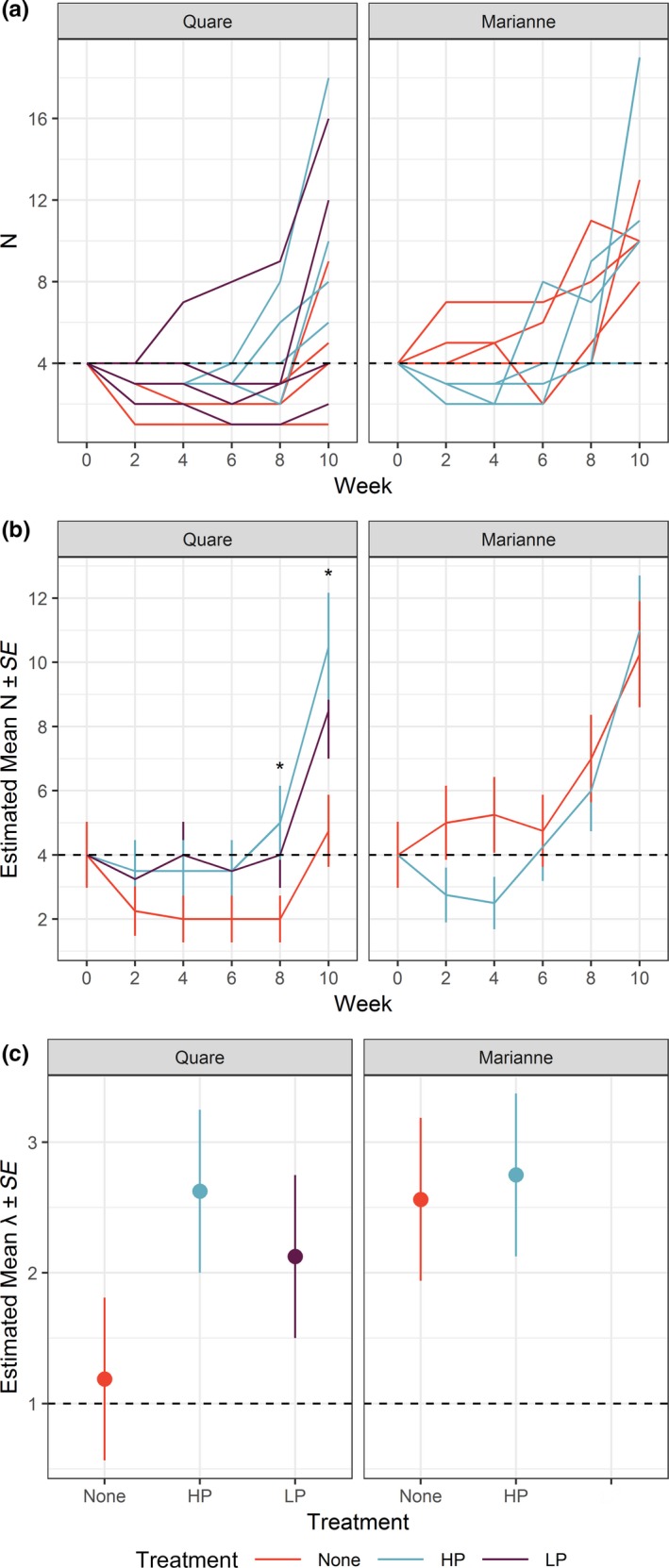
Population sizes (*N*) over time in each experimental tank (panel a), estimated marginal means and standard errors of population sizes over time (panel b), and estimated marginal means and standard errors of population growth rates (λ) between population founding and end of the experiment (panel c). Colors correspond to evolutionary history treatments: red, no gene flow (none); blue, gene flow from a high predation source (HP); purple, gene flow from a low predation source (LP). Horizontal dashed lines represent starting population size in panels a and b, and the value of *λ* indicating no population growth in panel c. Population sizes were significantly different only between the no gene flow and HP gene flow treatments (*p* < .05) at weeks 8 and 10 in Quare populations, as indicated with asterisks. There were no significant differences among population growth rates

Population sizes in individual tanks were highly variable but generally increased over time (Table 1; Figure 3a). According to pairwise comparisons of estimated marginal means, populations were significantly larger in the Quare HP gene flow treatment than in the no gene flow treatment at weeks 8 and 10 (*t*_75_ = 2.13, *p* = .0368 and t_75_ = 2.78, *p* = .0068, respectively; Figure 3b). The Quare LP gene flow treatment was nearly significantly larger than in the no gene flow treatment at week 10 (*t*_75_ = 1.97, *p* = .0523). Among Marianne populations, the no gene flow treatment increased relative to the HP gene flow treatment at first (nearly significant at week four; *t*_75_ = −1.87, *p* = .0648), but populations attained similar sizes by end of the experiment (Figure 3b). Estimated marginal means of finite population growth rates were similar and high in all treatments except for Quare no gene flow, which remained close to one, indicating no growth (Figure 3c).

### 3.3 Ecosystem effects

Linear mixed models and linear models used to test the effects of recent evolutionary history on ecosystem variables revealed no effect of gene flow treatment (HP, LP, none, and control) for either population on decomposition rates, benthic algae accumulation, chlorophyll‐α level in pelagic algae, or copepod/cladoceran zooplankton density. We did find a significant and positive correlation between total fish biomass and copepod density (*R* ^2^ = 2.084, F18 = 4.739, *p* = .04305). However, this relationship was heavily influenced by a single outlier and then became insignificant when the outlier was removed (*R* ^2^ = 0.04126, F17 = 0.7316, *p* = .4043).

## 4 DISCUSSION

Our study tested how populations with different recent evolutionary histories responded to a scenario of extreme population size reduction followed by a period of high environment quality. We uncovered fitness benefits in the form of individual growth from previous gene flow that only became apparent in high resource environments. We also investigated how evolutionary histories affected ecosystem dynamics. Our results suggest that recent evolutionary histories affect individual and sometimes population growth rates, but do not scale up to induce measurable effects on the ecosystem. Additional research is needed to fully parse out the relationship between evolutionary history and ecosystem dynamics.

### 4.1 Effects of evolutionary history on individual growth

When guppies were exposed to novel high resource environments, we found faster individual growth in females from populations that had previously received gene flow compared to those from populations without a history of gene flow; a result replicated in both Quare and Marianne drainages. There are several possible explanations for this result. One possibility is that certain traits of the original immigrant population (i.e., source of gene flow) facilitated higher growth in novel environments. In other words, if immigrants had inherently faster growth, it is possible that they conferred this trait to the recipient populations. Guppy populations show variation in individual growth with HP populations typically showing faster growth than LP populations (Reznick, Bryant, Reznick, & Bryant, 2007). In our study, if faster growth in individuals was caused by immigrants' traits, we would expect the HP gene flow treatments to grow faster than the LP treatments due to their fast life history (i.e., faster growth, more offspring, and earlier maturation times). However, we found individual growth were higher in all gene flow treatments, regardless of the environment from which the source population originated, suggesting that the observed growth advantage was not solely related to specific traits conferred by immigrants with a fast life history.

Another possible explanation is that individuals from populations with recent gene flow was able to grow faster on the same level of resources due to acquiring and metabolizing resources more effectively. A recent study found that brook trout with recent gene flow exhibited larger body sizes than brook trout that had not received gene flow while living in the same environments. This was possibly caused by overdominance due to heterosis, or increased fitness in hybrid offspring compared to parents, in somatic juvenile growth (Robinson et al., 2017). However, overdominance is unlikely to be the cause of treatment differences in our experiment because multiple generations of admixture in gene flow treatments had occurred prior to our study (Kronenberger et al., 2018). In Kronenberger et al. (2018), the authors genotyped every adult and reconstructed pedigrees to confirm the presence of hybridization. They found that Quare populations produced more hybrids than Marianne populations, but at least some admixture was observed in all gene flow treatments used as source populations for this study. Since treatments were left untampered with for over a year, it is unlikely that heterosis would have occurred in our experiment.

Another possibility is that higher overall genetic variation facilitated a growth benefit to individuals from the gene flow treatments. Genetic variation has been shown in many previous studies to have a positive effect on fitness (Reed, Frankham, Reed, & Frankham, 2016). For example, genetic diversity was positively correlated with mean lifetime expectancy in Chalkhill blue butterflies (Vandewoestijne, Schtickzelle, & Baguette, 2008), honey bee colonies with higher genetic diversity were able to out‐forage and gain weight faster than colonies with lower genetic diversity (Mattila & Seeley, 2007), and in blue mussels it was shown that individuals with higher heterozygosity also had higher average growth rates (Koehn & Gaffney, 1984). In the latter study, the authors proposed that faster growth rates in more heterozygous mussels were possible due to a greater availability of energy from the decreased costs of metabolism, specifically oxygen consumption rates. In our study, it is possible that fish with increased genetic variation due to gene flow were able to more efficiently utilize the high resources in outdoor mesocosm environments.

Regardless of the mechanism, the finding of significantly higher individual growth in both Quare and Marianne gene flow treatments is surprising in light of the results from Kronenberger et al. (2018), which documented consistent genetic rescue in Quare but not Marianne. The lack of consistent benefits of gene flow in Marianne treatments was previously interpreted as resulting from (a) higher overall initial levels of genetic variation in the recipient population and (b) relatively few generations of admixture over the course of the study. In Kronenberger et al. (2018), all tanks were fed a constant diet of fish flakes, guppies were constrained to 10‐gallon glass tanks, likely limiting population growth, and the indoor environment limited primary productivity. When the same guppies were moved to spacious, high resource outdoor mesocosms, individual growth was significantly higher in gene flow treatments from both Marianne and Quare drainages, suggesting some degree of fitness increase in Marianne guppies with previous gene flow that only became evident in a more favorable environment.

### 4.2 Effects of evolutionary history on population growth rates

Although population growth rates did not significantly differ among gene flow treatments, there was a trend for higher population growth rates in Quare gene flow treatments, but not Marianne. Similarly, we found that gene flow treatments achieved a significantly higher mean population size than no gene flow treatments in the Quare, but not Marianne. This discrepancy between the two drainages matches results from Kronenberger et al. (2018), namely, that gene flow was only consistently beneficial to recipient populations from Quare. As discussed in Kronenberger et al. (2018), Quare populations may have benefited more from the new variation provided by gene flow due to lower initial levels of genetic variation and small effective population sizes compared to the more diverse Marianne population. Importantly, the initial benefits of gene flow documented in Kronenberger et al. (2018) appear to have persisted for Quare populations with a history of gene flow in the novel and high resource environments of our study whereas, at the population level, gene flow did not appear to consistently benefit Marianne in either study. However, the large final population sizes reached by gene flow treatments from both drainages, and the fact that significant differences were detected among any treatments is especially striking given the severe bottleneck imposed at the beginning of the experiment (starting populations of two males and two females) and the short experimental duration of 10 weeks.

### 4.3 Effect of evolutionary history on ecosystem variables

In this study, the ecosystem variables we measured (zooplankton density, pelagic algae growth, benthic algae growth, and decomposition rates) did not significantly differ among any treatments (see Supporting Information). Fish density also did not appear to influence ecosystem variables although total fish biomass did have a significant but counterintuitive effect on copepod density where greater fish biomass was positively associated with copepod density. This could be due to an unmeasured indirect effect of guppies feeding on copepod predators such as macroinvertebrates. However, given that this result was dependent on a single outlier, we do not have confidence that it represents a meaningful relationship.

A possible explanation for the lack of differentiation among treatments is that algal and zooplankton communities were able to regenerate at rates fast enough to offset depletion by guppies. A previous study found that the population abundances of many zooplankton species were unaffected by fish predation and that only larger zooplankton species were susceptible to predation (Jack & Thorp, 2002). In our study, we quantified zooplankton density by order (i.e., cladoceran or copepod) and it is possible that a finer resolution of species composition data could have revealed differences among treatments. Also, previous studies have found that algal depletion by zooplankton predation can be offset by the nutrient release due to predation as algal communities use the nutrients to quickly regenerate (Sterner, 1986). However, these explanations do not explain the lack of differentiation of decomposition rates among treatments.

Another factor that could have contributed to these results is that macroinvertebrate species composition and abundance were not measured in tanks. This unmeasured component of the trophic system in our cattle tanks may have played an important role. For example, macroinvertebrates could have been an alternate food source for guppies or affected ecosystem variables and explained individual growth differences among treatments without changes in the resource levels we measured. The lack of differences in ecosystem measurements could also be explained by the relatively short time frame of our experiment or the large size of the cattle tanks with low numbers of fish, making the environments too large for such low fish densities to accrue any measurable changes on ecosystem factors. Our experiment consisted of relatively low replication with only four mesocosms per treatment and four fish per starting population. Given that ecosystem measurements are often highly stochastic, low replication also likely contributed to a lack of power to detect measurable ecosystem differences between treatments.

Interestingly, even though the ecosystem variables we measured were not different among treatments, individual guppy growth and population sizes did differ. This suggests a possible intrinsic mechanism that facilitated individuals from populations that previously received gene flow to grow faster on the same quality and quantity of resources. As discussed briefly above, this could be due to an inverse relationship between heterozygosity and the energetic costs of metabolism, possibly mediated by oxygen consumption. A relationship between heterozygosity and population growth rate has received mixed empirical support in other systems where a positive correlation has been found in American oysters (Koehn & Gaffney, 1984) and some populations of tiger salamanders (Pierce & Mitton, 1982), but not in European plaice (McAndrew, Ward, & Beardmore, 1986) or pink salmon (Beacham & Withler, 1985).

### 4.4 Environment‐dependent benefits of gene flow

Previous gene flow augmentation experiments have focused on fitness benefits that arise in deliberately challenging environments (Hufbauer et al., 2015) or potentially stressful laboratory environments (Kronenberger et al., 2018). While these results make important contributions to our growing understanding of how gene flow can rescue populations, it is also critical to understand how augmented populations will perform in “good” environments or periods of low environmental stress and change. In our study, we discovered consistent growth benefits resulting from gene flow in a high resource environment in populations that had not initially shown consistent fitness benefits. This result highlights the importance of using habitat improvement in combination with gene flow augmentation in wildlife management situations. If habitat enhancement and assisted gene flow are used in conjunction, the recipient population may have a better chance of long‐term persistence. Genetic rescue may only allow populations to reach larger population sizes and gain more genetic variation, both of which can help prevent extinction during periods of environmental stress, but may also facilitate faster population recovery when conditions are favorable.

### 4.5 Caveats, future directions, and broader implications

At first glance, it may seem biologically unrealistic to test eco‐evolutionary effects of a tropical fish in temperate ponds. However, guppies are a highly successful invasive species throughout the world—they encounter and thrive in a wide range of novel environments. Due to human introductions (accidental and intentional), guppies have established in sugarcane fields in Australia (Lindholm et al., 2005), artificially heated streams near power plants in Germany (Jourdan et al., 2014), and streams fed by hot springs in Japan (Shoji, Yokoyama, & Kawata, 2007). Although our study was carried out in lentic mesocosms in Michigan, as opposed to their native lotic Trinidadian streams, we think our results can be considered in the contexts of both invasive populations and rapid global change. For example, the lack of observable community effects in our study (in novel and high resource environments), compared with previous findings of effects of population history at the community level in native environments beckons for future work exploring eco‐evolutionary dynamics in native versus novel environments. Testing this question using fish densities that are more comparable to natural densities than the low densities in our experiment would be advisable.

Our study sets the stage for additional follow‐up research that will elucidate many remaining uncertainties. For example, future experiments that start with larger initial population sizes might better represent the genetic and phenotypic variation within each evolutionary history treatment and reduce the potentially confounding role of founder's effect. We imposed a strong founder's effect by seeding tanks with only four individuals (two males and two females) and this small sample size may not have represented the phenotypic and genetic variation present in the Kronenberger et al. (2018) treatments. To better characterize, the effect of recent evolutionary history on the environment, starting with larger fish densities or smaller mesocosms, may also create higher resource depletion rates and facilitate testing for significant differences in ecosystem variables. Future studies would also benefit by accounting for the composition and density of macroinvertebrates to understand how they affect other ecosystem variables and whether they serve as an alternate food source. Lastly, longer‐term monitoring of mesocosm populations and environments than what was possible here will provide a better opportunity to reveal treatment differences. Ecosystem‐level differences may become more detectable as resources are depleted over a longer study period.

As habitat fragmentation increases and native populations become smaller and more isolated, gene flow augmentation is a potentially powerful option to reverse the effects of inbreeding depression and genetic drift (Frankham et al., 2017). Previous work has shown that genetic rescue generally provides an immediate fitness increase to small populations (Whiteley et al., 2015; Frankham, 2016). In this study, we complement studies investigating the initial fitness effects of gene flow augmentation by considering longer‐term impacts of recent evolutionary history on individuals and populations in a benign environment. In addition, we investigate the impact of evolutionary history on ecosystem structure. The scenario we studied, in which populations become exposed to new conditions (first a severe bottleneck and then a novel, high resource environment), is highly plausible for contemporary populations—including ones that are not of conservation concern. For example, invasive species often experience a strong bottleneck when few individuals first colonize a new habitat and then are exposed to favorable conditions with many possible niches to exploit and few natural predators (Tsutsui, Suarez, Holway, & Case, 2002). Understanding recent evolutionary history, including nonselective forces like gene flow and genetic drift, may help to predict invasive potential. Additional research is needed in this area to fully understand the eco‐evolutionary and conservation implications of how augmented gene flow versus sustained genetic drift and inbreeding affects populations and ecosystems.

## CONFLICT OF INTEREST

None declared.

## AUTHOR CONTRIBUTION

S. W. F and M. L. M designed the study and collected data. J. A. K. and M. L. M. performed statistical analyses. All authors contributed to writing the paper.

## Data Availability

The datasets generated and analyzed in this study are available at https://doi.org/10.5061/dryad.fqz612jp8.

## References

[ece35879-bib-0001] Adams, J. R. , Vucetich, L. M. , Hedrick, P. W. , Peterson, R. O. , & Vucetich, J. A. (2011). Genomic sweep and potential genetic rescue during limiting environmental conditions in an isolated wolf population. Proceedings of the Royal Society B: Biological Sciences, 278(1723), 3336–3344. 10.1098/rspb.2011.0261 PMC3177630 21450731

[ece35879-bib-0002] Aitken, S. N. , & Whitlock, M. C. (2013). Assisted gene flow to facilitate local adaptation to climate change. Annual Review of Ecology, Evolution, and Systematics, 44(1), 367–388. 10.1146/annurev-ecolsys-110512-135747

[ece35879-bib-0003] Barson, N. J. , Cable, J. , & Van Oosterhout, C. (2009). Population genetic analysis of microsatellite variation of guppies (*Poecilia reticulata*) in Trinidad and Tobago: Evidence for a dynamic source‐sink metapopulation structure, founder events and population bottlenecks. Journal of Evolutionary Biology, 22(3), 485–497. 10.1111/j.1420-9101.2008.01675.x 19210594

[ece35879-bib-0004] Bassar, R. D. , Marshall, M. C. , López‐Sepulcre, A. , Zandonà, E. , Auer, S. K. , Travis, J. , … Reznick, D. N. (2010). Local adaptation in Trinidadian guppies alters ecosystem processes. Proceedings of the National Academy of Sciences of the United States of America, 107(8), 3616–3621. 10.1073/pnas.0908023107 20133670 PMC2840427

[ece35879-bib-0005] Bates, D. , & Maechler, M. (2009). lme4 linear mixed-effects models using S4 classes. R package version 0.999375-32.

[ece35879-bib-0006] Beacham, T. D. , & Withler, R. E. (1985). Heterozygosity and morphological variability of pink salmon (*Oncorhynchus gorbuscha*) from southern British Columbia and Puget Sound. Canadian Journal of Genetics and Cytology, 27(5), 571–579. 10.1139/g85-084

[ece35879-bib-0007] Benazzo, A. , Trucchi, E. , Cahill, J. A. , Maisano Delser, P. , Mona, S. , Fumagalli, M. , … Bertorelle, G. (2017). Survival and divergence in a small group: The extraordinary genomic history of the endangered Apennine brown bear stragglers. Proceedings of the National Academy of Sciences of the United States of America, 114(45), E9589–E9597. 10.1073/pnas.1707279114 29078308 PMC5692547

[ece35879-bib-0008] Burnham, K. P. , & Anderson, D. R. (2002). Model selection and multimodel inference (2nd ed.). New York, NY: Springer.

[ece35879-bib-0009] Carroll, S. P. , Hendry, A. P. , Reznick, D. N. , & Fox, C. W. (2007). Evolution on ecological time‐scales. Functional Ecology, 21(3), 387–393. 10.1111/j.1365-2435.2007.01289.x

[ece35879-bib-0010] Chevin, L. , Lande, R. , & Mace, G. M. (2010). Adaptation, plasticity, and extinction in a changing environment: Towards a predictive theory. PLoS Biology, 8(4), 10.1371/journal.pbio.1000357 PMC2864732 20463950

[ece35879-bib-0011] Crutsinger, G. M. , Collins, M. D. , Fordyce, J. A. , Gompert, Z. , Nice, C. C. , & Sanders, N. J. (2006). An ecosystem process. Cell, 647(August), 966–968. 10.1126/science.1128326 16917062

[ece35879-bib-0012] Edmands, S. (2007). Between a rock and a hard place: Evaluating the relative risks of inbreeding and outbreeding for conservation and management. Molecular Ecology, 16(3), 463–475. 10.1111/j.1365-294X.2006.03148.x 17257106

[ece35879-bib-0013] Ezard, T. H. G. , Côté, S. D. , & Pelletier, F. (2009). Eco‐evolutionary dynamics: Disentangling phenotypic, environmental and population fluctuations. Philosophical Transactions of the Royal Society B: Biological Sciences, 364(1523), 1491–1498. 10.1098/rstb.2009.0006 PMC2690502 19414464

[ece35879-bib-0014] Fitzpatrick, S. W. , Gerberich, J. C. , Angeloni, L. M. , Bailey, L. L. , Broder, E. D. , Torres‐Dowdall, J. , … Funk, C. W. (2016). Gene flow from an adaptively divergent source causes rescue through genetic and demographic factors in two wild populations of Trinidadian guppies. Evolutionary Applications, 9(7), 879–891. 10.1111/eva.12356 27468306 PMC4947150

[ece35879-bib-0015] Fitzpatrick, S. W. , Gerberich, J. C. , Kronenberger, J. A. , Angeloni, L. M. , & Funk, W. C. (2015). Locally adapted traits maintained in the face of high gene flow. Ecology Letters, 18(1), 37–47. 10.1111/ele.12388 25363522

[ece35879-bib-0016] Frankham, R. (2015). Genetic rescue of small inbred populations: Meta‐analysis reveals large and consistent benefits of gene flow. Molecular Ecology, 24(11), 2610–2618. 10.1111/mec.13139 25740414

[ece35879-bib-0017] Frankham, R. (2016). Genetic rescue benefits persist to at least the F3 generation, based on a metaanalysis. Biological Conservation, 195, 33–36. 10.1016/j.biocon.2015.12.038

[ece35879-bib-0018] Frankham, R. , Ballou, J. D. , Ralls, K. , Eldridge, M. D. , Dudash, M. R. , Fenster, C. B. , … Sunnucks, P. (2017). Genetic management of fragmented animal and plant populations. Oxford, UK: Oxford University Press.

[ece35879-bib-0019] Ghalambor, C. K. , McKay, J. K. , Carroll, S. P. , & Reznick, D. N. (2007). Adaptive versus non‐adaptive phenotypic plasticity and the potential for contemporary adaptation in new environments. Functional Ecology, 21(3), 394–407. 10.1111/j.1365-2435.2007.01283.x

[ece35879-bib-0020] Gilpin, M. E. , & Soule, M. E. (1983). Minimum viable populations: processes of species extinction In Conservation biology: The science of scarcity and diversity (pp. 19–34). Sunderland, MA: Sinauer Associates.

[ece35879-bib-0021] Grant, P. R. (1986). Ecology and evolution of Darwin's finches. Princeton, NJ: Princeton University Press.

[ece35879-bib-0022] Hairston, N. G. , Ellner, S. P. , Geber, M. A. , Yoshida, T. , & Fox, J. A. (2005). Rapid evolution and the convergence of ecological and evolutionary time. Ecology Letters, 8(10), 1114–1127. 10.1111/j.1461-0248.2005.00812.x

[ece35879-bib-0023] Harmon, L. J. , Matthews, B. , Des Roches, S. , Chase, J. M. , Shurin, J. B. , & Schluter, D. (2009). Evolutionary diversification in stickleback affects ecosystem functioning. Nature, 458(7242), 1167–1170. 10.1038/nature07974 19339968

[ece35879-bib-0026] Hogg, J. T. , Forbes, S. H. , Steele, B. M. , & Luikart, G. (2006). Genetic rescue of an insular population of large mammals. Proceedings of the Royal Society B: Biological Sciences, 273(1593), 1491–1499. 10.1098/rspb.2006.3477 PMC1560318 16777743

[ece35879-bib-0027] Hufbauer, R. A. , Szűcs, M. , Kasyon, E. , Youngberg, C. , Koontz, M. J. , Richards, C. , … Melbourne, B. A. (2015). Three types of rescue can avert extinction in a changing environment. Proceedings of the National Academy of Sciences of the United States of America, 112(33), 10557–10562. 10.1073/pnas.1504732112 26240320 PMC4547288

[ece35879-bib-0028] Hughes, A. R. , Inouye, B. D. , Johnson, M. T. J. , Underwood, N. , & Vellend, M. (2008). Ecological consequences of genetic diversity. Ecology Letters, 11(6), 609–623. 10.1111/j.1461-0248.2008.01179.x 18400018

[ece35879-bib-0029] Jack, J. D. , & Thorp, J. H. (2002). Impacts of fish predation on an Ohio River zooplankton community. Journal of Plankton Research, 24(2), 119–127. 10.1093/plankt/24.2.119

[ece35879-bib-0030] Johnson, A. M. , Chappell, G. , Price, A. C. , Helen Rodd, F. , Olendorf, R. , & Hughes, K. A. (2010). Inbreeding depression and inbreeding avoidance in a natural population of guppies (*Poecilia reticulata*). Ethology, 116(5), 448–457. 10.1111/j.1439-0310.2010.01763.x

[ece35879-bib-0031] Johnson, W. E. , Onorato, D. P. , Roelke, M. E. , Land, E. D. , Cunningham, M. , Belden, R. C. , … O'Brien, S. J. (2010). Genetic restoration of the Florida panther. Science, 329(5999), 1641–1645. 10.1126/science.1192891 20929847 PMC6993177

[ece35879-bib-0032] Jourdan, J. , Miesen, F. W. , Zimmer, C. , Gasch, K. , Herder, F. , Schleucher, E. , … Bierbach, D. (2014). On the natural history of an introduced population of guppies (*Poecilia reticulata* Peters, 1859) in Germany. BioInvasions Records, 3(3), 175–184. 10.3391/bir.2014.3.3.07

[ece35879-bib-0033] Kinnison, M. T. , & Hairston, N. G. (2007). Eco‐evolutionary conservation biology: Contemporary evolution and the dynamics of persistence. Functional Ecology, 21(3), 444–454. 10.1111/j.1365-2435.2007.01278.x

[ece35879-bib-0034] Koehn, R. K. , & Gaffney, P. M. (1984). Genetic heterozygosity and growth rate in *Mytilus edulis* . Marine Biology, 82(1), 1–7. 10.1007/BF00392757

[ece35879-bib-0035] Kronenberger, J. A. , Gerberich, J. C. , Fitzpatrick, S. W. , Broder, E. D. , Angeloni, L. M. , & Funk, W. C. (2018). An experimental test of alternative population augmentation scenarios. Conservation Biology, 32(4), 838–848. 10.1111/cobi.13076 29349820

[ece35879-bib-0036] Lande, R. (1988). Genetics and biological demography in conservation. Science, 241(4872), 1455–1460. 3420403 10.1126/science.3420403

[ece35879-bib-0037] Lindholm, A. K. , Breden, F. , Alexander, H. J. , Chan, W. K. , Thakurta, S. G. , & Brooks, R. (2005). Invasion success and genetic diversity of introduced populations of guppies *Poecilia reticulata* in Australia. Molecular Ecology, 14(12), 3671–3682. 10.1111/j.1365-294X.2005.02697.x 16202088

[ece35879-bib-0038] Lowe, W. H. , Kovach, R. P. , & Allendorf, F. W. (2017). Population genetics and demography unite ecology and evolution. Trends in Ecology and Evolution, 32(2), 141–152. 10.1016/j.tree.2016.12.002 28089120

[ece35879-bib-0039] Magurran, A. E. (2005). Evolutionary ecology: The Trinidadian guppy. Oxford, UK: Oxford University Press.

[ece35879-bib-0040] Mattila, H. R. , & Seeley, T. D. (2007). Genetic diversity in honey productivity and fitness. Science, 317(5836), 10–13. 10.1126/science.1143046 17641199

[ece35879-bib-0041] McAndrew, B. J. , Ward, R. D. , & Beardmore, J. A. (1986). Growth rate and heterozygosity in the plaice, pleuronectes. Heredity, 57(2), 171–180. 10.1038/hdy.1986.107 7076510

[ece35879-bib-0044] Pelletier, F. , Garant, D. , & Hendry, A. P. (2009). Eco‐evolutionary dynamics. Philosophical Transactions of the Royal Society B: Biological Sciences, 364(1523), 1483–1489. 10.1098/rstb.2009.0027 PMC2690510 19414463

[ece35879-bib-0045] Pierce, B. A. , & Mitton, J. B. (1982). Allozyme heterozygosity and growth in the tiger salamander, *Ambystoma tigrinum* . Journal of Heredity, 73(4), 250–253. 10.1093/oxfordjournals.jhered.a109633 7108182

[ece35879-bib-0046] Post, D. M. , & Palkovacs, E. P. (2009). Eco‐evolutionary feedbacks in community and ecosystem ecology: Interactions between the ecological theatre and the evolutionary play. Philosophical Transactions of the Royal Society B: Biological Sciences, 364(1523), 1629–1640. 10.1098/rstb.2009.0012 PMC2690506 19414476

[ece35879-bib-0047] Reed, D. H. , Frankham, R. , Reed, D. H. , & Frankham, R. (2016). Correlation between fitness and genetic diversity correlation between fitness and genetic diversity. Conservation Biology, 17(1), 230–237. 10.1046/j.1523-1739.2003.01236.x

[ece35879-bib-0048] Reznick, D. , Bryant, M. , Reznick, D. , & Bryant, M. (2007). Comparative long‐term mark‐recapture studies of guppies (*Poecilia reticulata*): Differences among high and low predation localities in growth and survival. Finnish Zoological and Botanical Publishing Board, 44(2), 152–160.

[ece35879-bib-0049] Reznick, D. N. , Butler, M. J. IV , Rodd, F. H. , & Ross, P. (1996). Life‐history evolution in guppies (*Poecilia reticulata*). 6. Differential mortality as a mechanism for natural selection. Evolution, 50, 1651–1660. 28565709 10.1111/j.1558-5646.1996.tb03937.x

[ece35879-bib-0050] Reznick, D. , & Endler, J. A. (1982). The impact of predation on life history evolution. Evolution, 36(1), 160–177. 28581096 10.1111/j.1558-5646.1982.tb05021.x

[ece35879-bib-0051] Robinson, Z. L. , Coombs, J. A. , Hudy, M. , Nislow, K. H. , Letcher, B. H. , & Whiteley, A. R. (2017). Experimental test of genetic rescue in isolated populations of brook trout. Molecular Ecology, 26(17), 4418–4433. 10.1111/mec.14225 28664980

[ece35879-bib-0052] Rudolf, V. H. W. , & Rasmussen, N. L. (2013). Ontogenetic functional diversity: Size structure of a keystone predator drives functioning of a complex ecosystem. Ecology, 94(5), 1046–1056. 10.1890/12-0378.1 23858645

[ece35879-bib-0053] Saccheri, I. , Kuussaari, M. , Kankare, M. , Vikman, P. , Fortelious, W. , & Hanski, I. (1998). Inbreeding and extinction in a butterfly metapopulation. Nature, 392, 491–494. Retrieved from http://www.davidsbatista.net/blog/2017/11/13/Conditional_Random_Fields

[ece35879-bib-0054] Shoji, A. , Yokoyama, J. , & Kawata, M. (2007). Molecular phylogeny and genetic divergence of the introduced populations of Japanese guppies, *Poecilia reticulata* . Conservation Genetics, 8(2), 261–271. 10.1007/s10592-006-9166-1

[ece35879-bib-0055] Slobodkin, L. B. (1961). Growth and regulation of animal populations. New York, NY: Holt, Rinehart and Winston.

[ece35879-bib-0056] Smallbone, W. , van Oosterhout, C. , & Cable, J. (2016). The effects of inbreeding on disease susceptibility: *Gyrodactylus turnbulli* infection of guppies*, Poecilia reticulata* . Experimental Parasitology, 167, 32–37. 10.1016/j.exppara.2016.04.018 27130704

[ece35879-bib-0057] Sterner, R. W. (1986). Herbivores' direct and indirect effects on algal populations. Science, 231(4738), 605–607. 17750971 10.1126/science.231.4738.605

[ece35879-bib-0058] Swindell, W. R. , & Bouzat, J. L. (2006). Gene flow and adaptive potential in *Drosophila melanogaster* . Conservation Genetics, 7(1), 79–89. 10.1007/s10592-005-8223-5

[ece35879-bib-0059] Tallmon, D. A. , Luikart, G. , & Waples, R. S. (2004). The alluring simplicity and complex reality of genetic rescue. Trends in Ecology and Evolution, 19(9), 489–496. 10.1016/j.tree.2004.07.003 16701312

[ece35879-bib-0060] Tsutsui, N. D. , Suarez, A. V. , Holway, D. A. , & Case, T. J. (2002). Reduced genetic variation and the success of an invasive species. Proceedings of the National Academy of Sciences of the United States of America, 97(11), 5948–5953. 10.1073/pnas.100110397 PMC18539 10811892

[ece35879-bib-0061] Van Oosterhout, C. , Trigg, R. E. , Carvalho, G. R. , Magurran, A. E. , Hauser, L. , & Shaw, P. W. (2003). Inbreeding depression and genetic load of sexually selected traits: How the guppy lost its spots. Journal of Evolutionary Biology, 16(2), 273–281. 10.1046/j.1420-9101.2003.00511.x 14635866

[ece35879-bib-0062] Vandewoestijne, S. , Schtickzelle, N. , & Baguette, M. (2008). Positive correlation between genetic diversity and fitness in a large, well‐connected metapopulation. BMC Biology, 6, 1–11. 10.1186/1741-7007-6-46 18986515 PMC2587462

[ece35879-bib-0063] Weeks, A. R. , Heinze, D. , Perrin, L. , Stoklosa, J. , Hoffmann, A. A. , van Rooyen, A. , … Mansergh, I. (2017). Genetic rescue increases fitness and aids rapid recovery of an endangered marsupial population. Nature Communications, 8(1), 1–6. 10.1038/s41467-017-01182-3 PMC5715156 29057865

[ece35879-bib-0064] Westemeier, R. L. , Brawn, J. D. , Simpson, S. A. , Esker, T. L. , Jansen, R. W. , Walk, J. W. , … Paige, K. N. (1998). Tracking the long‐term decline and recovery of an isolated population. Science, 282(5394), 1695–1698. 10.1126/science.282.5394.1695 9831558

[ece35879-bib-0065] Whiteley, A. R. , Fitzpatrick, S. W. , Funk, W. C. , & Tallmon, D. A. (2015). Genetic rescue to the rescue. Trends in Ecology & Evolution, 30(1), 42–49. 10.1016/j.tree.2014.10.009 25435267

[ece35879-bib-0066] Yoshida, T. , Jones, L. E. , Ellner, S. P. , Fussmann, G. F. , & Hairston, N. G. (2003). Rapid evolution drives ecological dynamics in a predator – prey system. Nature, 424(July), 303–306. 10.1038/nature01767 12867979

