# Supplementary material for: Recent evolutionary history predicts population but not ecosystem‐level patterns
Source: Ecol Evol. 2019 Nov 28;9(24):14442–52. doi: 10.1002/ece3.5879 (PMC6953670; doi:10.1002/ece3.5879)
Supplement: Supplementary file 1 [file ECE3-9-14442-s001.docx]

**SUPPLEMENTAL DATA**

**Table S1.** The results of linear mixed models including each ecosystem variable, treatment type (no gene flow, HP gene flow, LP gene flow, and control) as a fixed effect and tank location as a random effect. Each treatment was tested against the null model that included only the random effect. More information on models is in the main text. For each model we report the R^2^, degrees of freedom, *F* and *p* values.

| **Ecosystem variable** | **R^2^** | **df** | ***F*** | ***p*** |
| --- | --- | --- | --- | --- |
| Leaf decomposition | 0.2211 | 18 | 1.069 | 0.41 |
| Pelagic algae | 0.1768 | 18 | 0.7734 | 0.5813 |
| Benthic algae | 0.1063 | 18 | 0.428 | 0.8232 |
| Cladoceran density | 0.1326 | 15 | 0.5734 | 0.6861 |
| Copepod density | 0.1246 | 15 | 0.5337 | 0.7131 |

**Table S2.** The results of simple linear regressions to correlate total fish biomass with each environmental variable at the last sampling period. For each model we report degrees of freedom, R^2^, *F*, and *p* values.

| **Ecosystem variable** | **Guppy population variable** | **R^2^** | **df** | ***F*** | ***p*** |
| --- | --- | --- | --- | --- | --- |
| Leaf decomposition | Fish biomass | 0.01391 | 22 | 0.3104 | 0.583 |
|  | Fish abundance | 0.001695 | 22 | 0.03735 | 0.8485 |
| Pelagic algae | Fish biomass | 0.02687 | 22 | 0.6076 | 0.444 |
|  | Fish abundance | 0.0002568 | 22 | 0.005651 | 0.9408 |
| Benthic algae | Fish biomass | 0.002692 | 22 | 0.05938 | 0.8097 |
|  | Fish abundance | 0.002984 | 22 | 0.06585 | 0.7999 |
| Cladoceran density | Fish biomass | 0.003645 | 18 | 0.06584 | 0.8004 |
|  | Fish abundance | 3.163e-05 | 18 | 0.0005693 | 0.9812 |
| Copepod density | Fish biomass | 0.2084 | 18 | 4.739 | 0.04305 |
|  | Fish biomass (outlier removed) | 0.04126 | 17 | 0.7316 | 0.4043 |
|  | Fish abundance | 0.05345 | 18 | 1.1016 | 0.3267 |


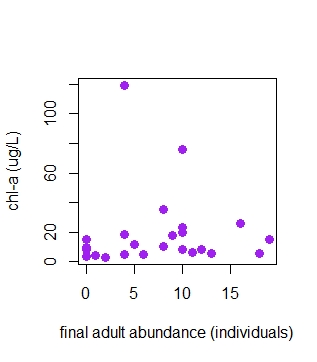

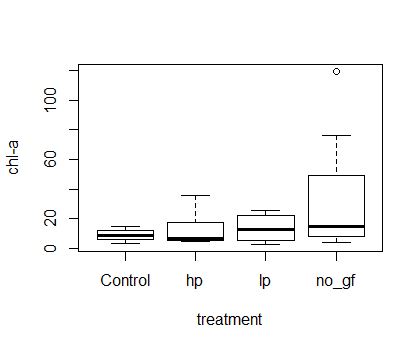


**
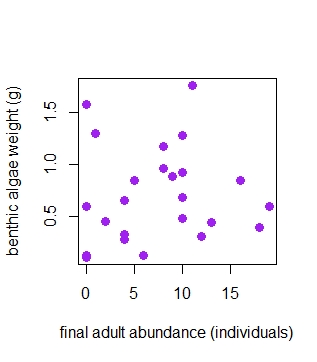

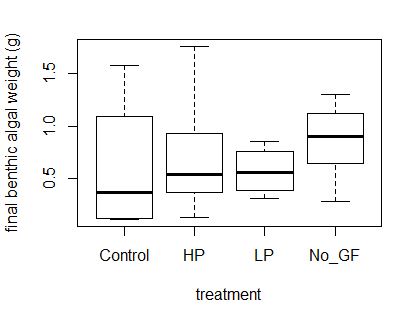
Figure S1**. Effect of gene flow treatments (a.) and final adult guppy abundance (b.) on chl-a (µg/L)

**Figure S2**. Effect of gene flow treatments (a.) and final adult guppy abundance (b.) on benthic algae weight (g)

**
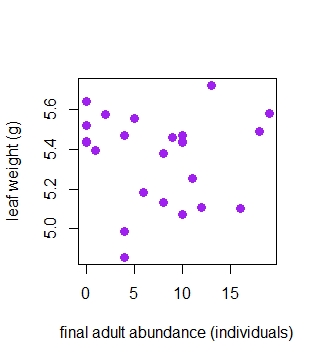

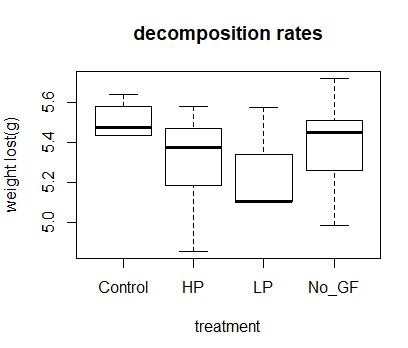
Figure S3**. Effect of gene flow treatments (a.) and final guppy abundance on (b.) on decomposition rates, listed here as total weight lost (g) from leaf litter mass


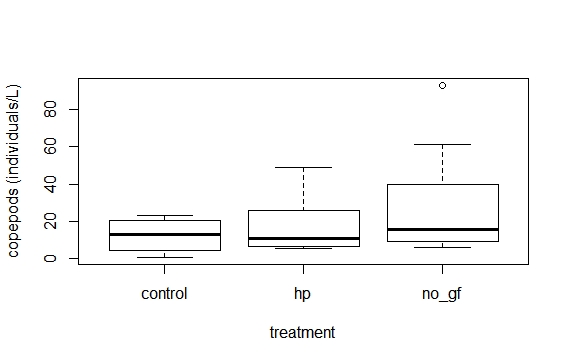

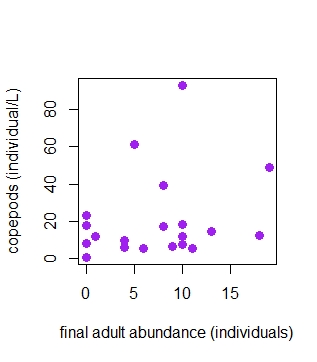


**Figure S4**. Effect of gene flow treatments (a.) and final guppy abundance on (b.) copepod density (individuals/L)


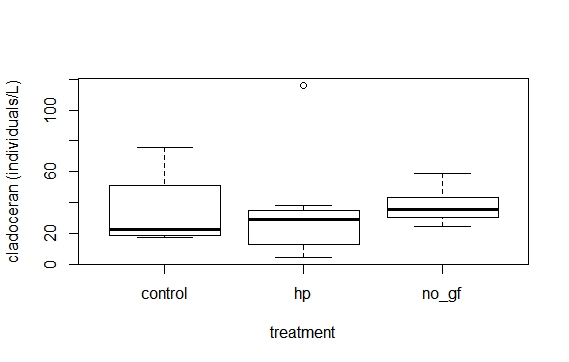

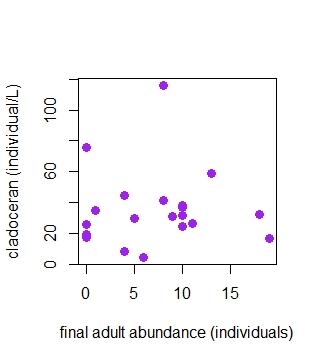


**Figure S5**. Effect of gene flow treatments (a.) and final guppy abundance on (b.) cladoceran density (individuals/L)
